# An update on excess mortality in the second year of the COVID-19 pandemic in Germany

**DOI:** 10.1007/s11943-022-00303-9

**Published:** 2022-03-15

**Authors:** Giacomo De Nicola, Göran Kauermann

**Affiliations:** Ludwig-Maximilians-Universität München, Munich, Germany

**Keywords:** COVID-19, Excess mortality, Expected mortality, Standardized mortality rate, COVID-19, Übersterblichkeit, Erwartete Sterblichkeit, Standardisierte Mortalitätsrate

## Abstract

In this short note, we apply the method of De Nicola et al. (2022) to the most recent available data, thereby providing up-to-date estimates of all-cause excess mortality in Germany for 2021. The analysis reveals a preliminary excess mortality of approximately 2.3% for the calendar year considered. The excess is mainly driven by significantly higher excess mortality in the 60-79 age group.

## Abstract (de)

In diesem kurzen Beitrag wenden wir die Methode von De Nicola et al. (2022) auf die neuesten verfügbaren Daten an und zeigen aktuelle Schätzungen der Gesamt-Übersterblichkeit in Deutschland für das Jahr 2021. Die Analyse zeigt eine vorläufige Übersterblichkeit von etwa 2,3% für das betrachtete Kalenderjahr. Dieser Wert ist hauptsächlich auf eine deutlich höhere Übersterblichkeit in der Altersgruppe der 60-79-Jährigen zurückzuführen.

In our article (De Nicola et al. 2022) in this issue, we presented a simple and novel method to compute excess mortality in a given calendar year while effectively taking the age structure of the population into account. We then applied our method to age-stratified mortality data to obtain estimates for general and age group-specific excess mortality for Germany in 2020, the first year of the COVID-19 pandemic. As we enter 2022, mortality figures from 2021 are starting to become available. With this short note, we thereby aim to provide the reader with up-to-date estimates of excess mortality for the second consecutive year of the pandemic. Mortality data are provided by the German Federal Statistical Office (Destatis 2022). Figures for 2021 are, at time point of submission of this note, not final, and numbers will presumably increase due to data corrections. We leave this problem aside here, and work with data as of February 1, 2022.

Fig. 1 gives an overview of the results for all age groups combined. We plot the expected death counts for each year as blue squares (see De Nicola et al. 2022 for details), and the observed death counts as black dots. We can see that overall excess mortality in 2021 was more pronounced than in 2020. More specifically, as of February 1, 2022, a total of 1 019 809 deaths were registered in Germany for the year 2021, i.e. 23 399 deaths more than expected. This corresponds to an estimated overall excess mortality of approximately 2.3%.

**Fig. 1:**
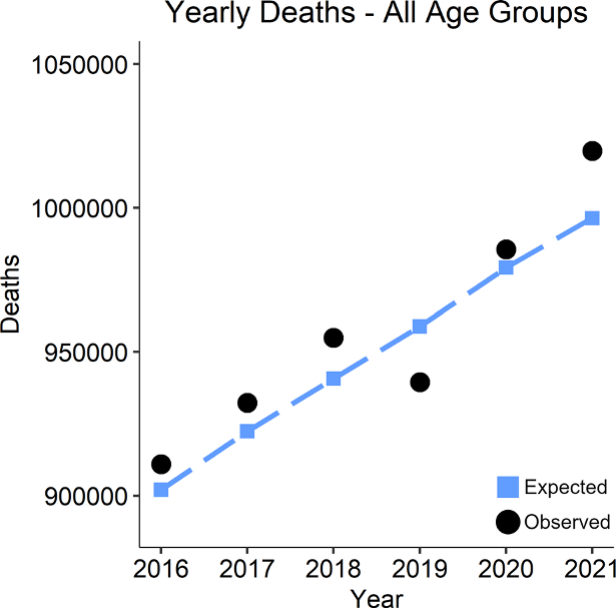
Expected deaths by year, represented by blue squares, plotted against observed fatalities, depicted by black dots. Overall excess mortality in 2021 was more pronounced than in 2020

Table 1 and Fig. 2 give a more complete picture of the mortality observed in 2021 for the different age groups. We observe that the most pronounced relative excess mortality was observed in the age groups 40–50, 60–70 and 70–80. We can also see how, in general, excess mortality was more driven by deaths in the 60–79 age category rather than in the 80+ group.

**Table 1:** Expected and observed yearly mortality in 2021 for each age group

Age group	Expected 2021	Observed 2021	Absolute diff.	Relative diff.
$$[00,30)$$	7383	7386	3	$$+0$$%
$$[30,40)$$	6696	6910	214	$$+3$$%
$$[40,50)$$	15 107	16 190	1083	$$+7$$%
$$[50,60)$$	58 593	59 221	628	$$+1$$%
$$[60,70)$$	120 356	126 183	5827	$$+5$$%
$$[70,80)$$	193 669	203 732	10 063	$$+5$$%
$$[80,90)$$	397 875	396 578	$$-1297$$	$$-0$$%
$[90,\infty)$	196 878	203 609	6731	$+3$%
Total	996 410	1 019 809	23 399	$+2$%

**Fig. 2:**
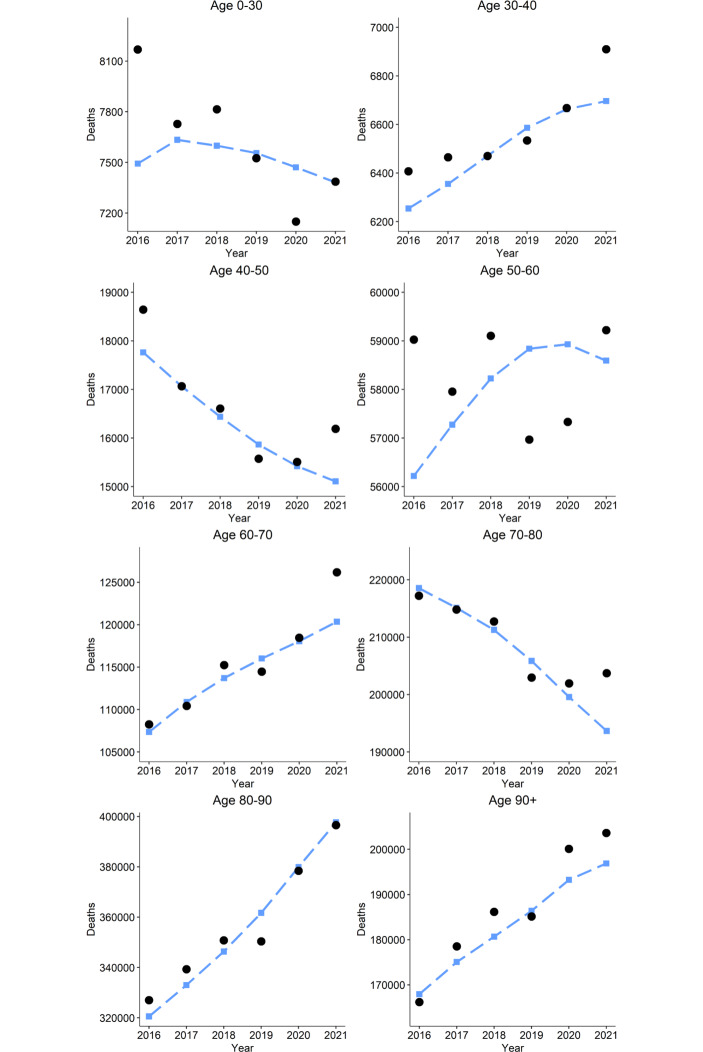
Expected deaths per year, represented by blue squares, plotted against observed fatalities, depicted by black dots, shown separately for each age group. Relative excess mortality in 2021 was most pronounced in the 40–50, 60–70 and 70–80 age categories

As a concluding note, we emphasise that all results presented here are based on provisional data, as the final death tolls for 2021 in Germany are not yet available at the time of writing. We can therefore expect some more deaths to be registered in the coming months. Based on past experience, those late registration should produce an increase of a few thousand units in the final toll (last year 982 489 deaths were registered for 2020 as of January 29, 2021, while the final, official toll amounted to 985 572). All in all, we can conclude that excess mortality for 2021 in Germany can, with data up to February 1, 2022, be estimated at a minimum of 2.3%, and that the final estimate will most likely be higher by a few decimal points.
